# Neanderthal Use of Fish, Mammals, Birds, Starchy Plants and Wood 125-250,000 Years Ago

**DOI:** 10.1371/journal.pone.0023768

**Published:** 2011-08-24

**Authors:** Bruce L. Hardy, Marie-Hélène Moncel

**Affiliations:** 1 Department of Anthropology, Kenyon College, Gambier, Ohio, United States of America; 2 Département de Préhistoire, Muséum National d'Histoire Naturelle, Institut de Paléontologie Humaine, Paris, France; University of Oxford, United Kingdom

## Abstract

Neanderthals are most often portrayed as big game hunters who derived the vast majority of their diet from large terrestrial herbivores while birds, fish and plants are seen as relatively unimportant or beyond the capabilities of Neanderthals. Although evidence for exploitation of other resources (small mammals, birds, fish, shellfish, and plants) has been found at certain Neanderthal sites, these are typically dismissed as unusual exceptions. The general view suggests that Neanderthal diet may broaden with time, but that this only occurs sometime after 50,000 years ago. We present evidence, in the form of lithic residue and use-wear analyses, for an example of a broad-based subsistence for Neanderthals at the site of Payre, Ardèche, France (beginning of MIS 5/end of MIS 6 to beginning of MIS 7/end of MIS 8; approximately 125–250,000 years ago). In addition to large terrestrial herbivores, Neanderthals at Payre also exploited starchy plants, birds, and fish. These results demonstrate a varied subsistence already in place with early Neanderthals and suggest that our ideas of Neanderthal subsistence are biased by our dependence on the zooarchaeological record and a deep-seated intellectual emphasis on big game hunting.

## Introduction

Meat, particularly in the form of hunting large game, has long been viewed as a vital component of human evolution as an energy rich food and valuable protein source [1]. Neanderthals, according to recent dietary reconstructions, have taken this adaptation to another level, deriving the vast majority of their calories from meat of large terrestrial herbivores [2]. Neanderthal sites certainly contain plenty of evidence of consumption of large herbivores, but there is increasing evidence that they also consumed small game, birds, fish, molluscs, and plants [3], [4]. Despite this evidence, the Neanderthal diet is still seen as consisting predominately of large herbivore meat. Many have argued that a diet such as this would lead to problems with protein poisoning and that some other energy source would have been necessary [1], [5]. The continued dominance of the Neanderthals as top carnivores hypothesis, even with the growing evidence that other types of game and plants were also consumed, speaks to the persistence and embedded nature of the big game hunting paradigm. Furthermore, it is unlikely that there was one Neanderthal diet, rather, diets likely varied according to the locally available resources [3], [6]. Here, we report evidence for consumption of a broad range of plant and animal foods by Neanderthals in interglacial contexts at the site of Payre in southern France 125–250 ka.

### The Site of Payre

**Figure 1:**
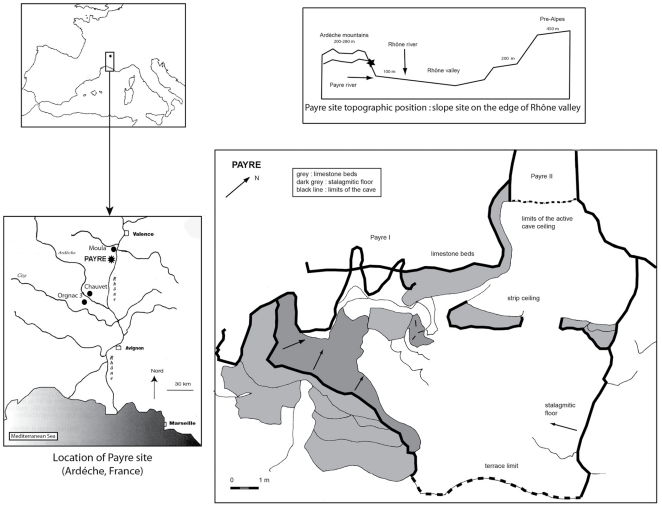
Location of the site of Payre.

**Figure 2:**
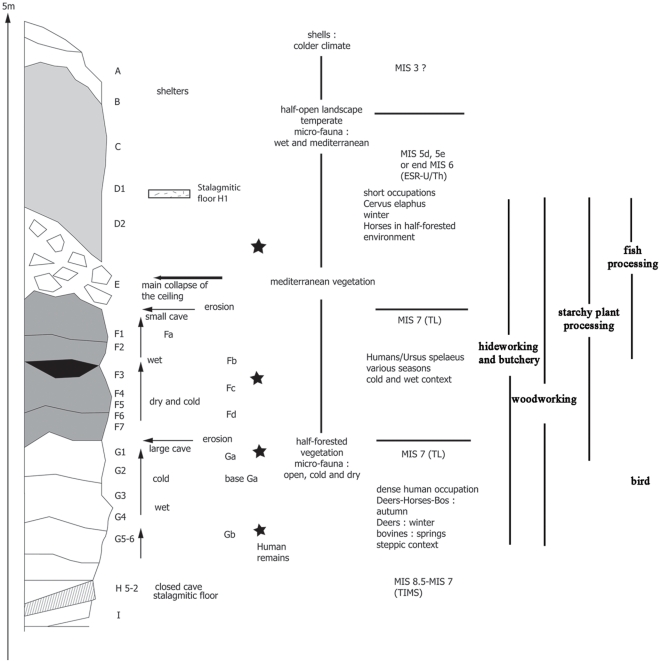
Stratigraphy, dating, climate, season, and activities at Payre.

The site of Payre is located in the Rhone Valley of France (Fig. 1). The site was first a cave, then a shelter before the collapse of the limestone ceiling. In spite of the varied nature of the site, Neanderthals came back at several different periods, perhaps because of its location on a promontory above the Rhone and Payre Valleys providing access to diverse environments. The excavations took place between 1990 and 2002 and yielded a 5 m thick sequence of deposits and 8 occupation levels. According to ESR, U-Th series, TL and TIMS methods, the sequence is dated to the end of MIS 8 and beginning of MIS 7 (levels Gb to Fa) and the end of MIS 6 and beginning of MIS 5 (levels E and D, Fig. 2) [7], [8]. Neanderthal remains were discovered throughout the sequence, but most of them are located in levels Gb and Ga. The lithic and faunal assemblages are related to the early Middle Paleaeolithic and Neanderthals came for short-term seasonal occupations [9]–[11]. These occupations took place under temperate conditions at the beginning of interglacial periods, as attested by the faunal, microfaunal and palynological studies [12]–[13]. Flint came from local and semi-local outcrops located on the southern plateau and geological surveys suggest that raw material gathering took place during other subsistence activities from various outcrops with some long distance transport of flint up to 60 km [14]–[15]. The core technology is mainly discoid on flint, secondarily on quartz and limestone. Discoid cores show one or two secant flaking surfaces (convex or pyramidal section of each surface) with centripetal or unidirectional removals. All stages of the lithic reduction sequence are present at the site for local and semi-local flint, and partial for local quartz. This kind of technology produces many diverse flakes (thin, thick, short, elongated, triangular or quandrangular). The main flake-tools are scrapers and points (around 10–15% for the whole series). Some large bifacial tools were worked outside the site on large quartzite and basalt pebbles found in the Rhone Valley bank.

Based on zooarchaeological analyses and skeletal part representation, three large herbivores were mainly hunted (cervids, equids and bovids). Whole carcasses of cervids were brought to the site. Rare remains of rhinoceros and elephants (limb and skull elements) suggest scavenging of these very large mammals. Bones are largely broken for marrow and cut-marks attest to human butchery activities. Fire is also in evidence through burned bones and flint and a small ash lens in level Ga. Hyenas and bear remains demonstrate that the cave was occasionally inhabited by carnivores, who came when Neanderthals left the cave, especially level F [7].

## Materials and Methods

### Lithic Use-wear and Residue Analysis

A sample of 182 minimally handled stone artifacts was examined under bright field incident light at magnifications ranging from 50–1000x using an Olympus BH30 microscope. Use-wear and adhering residues were photographed with a Nikon Coolpix 995 digital camera and their locations recorded on a line drawing of the artifact. Use-wear analysis included the identification of striations, edge rounding and microflake scarring to help identify relative hardness of the use-material and the use-action [16]–[17]. Due to the potential overlap of polishes from different worked materials, polishes were identified as either “soft” or “hard/high silica” [18]–[21]. Soft polish derives from working soft materials such as animal skin or muscle while hard/high silica polish forms from processing bone, antler, wood, or soft plants with high silica content. One additional category is characterized by dull greasy polish in linear streaks with bright spots and may be associated with fish processing [22]–[23].

Examination of residues on stone tool surfaces allows the identification of hair, feathers, animal tissue, bone/antler, starch grains, plant tissue, raphides, phytoliths, wood and resin [19], [24], [25]. Residues were identified based on comparison with modern experimental samples and published materials [26]–[41]. Fish processing experiments and comparison with histology of fish tissues [42] allowed the characterization of fish residues (nerve tissue, bone, skeletal muscle, epithelial tissue, iridophores, scales, etc.) [43]. Starch grains can potentially be mistaken for fungal spores [44]. In order to confirm starch identification, putative starch grains were extracted and observed under transmitted light [45]. Patterning and distribution of residues as well as the co-occurrence of use-wear helped establish that residues were related to use [17], [24], [46]–[47].

## Results

Of the 182 artifacts examined, 125 (68.7%) preserved some type of functional evidence. The results show processing of plants, wood, fish, bird, starchy plants, bone, butchery and hideworking. Some artifacts showed evidence for processing of multiple residue types. See Table 1 for a breakdown of activities by level.

**Table 1:** Frequency of worked materials by level.*

Level	Bone	Bird	Hide/Animal	Wood	HHSPlant	SoftPlant	StarchyPlant	Fish	Hard	Soft	Unknown
Gbn = 15	6.7%	---	33.3%	13.3%	20%	---	---	---	6.7%	---	33.3%
Gan = 16	---	6.3%	18.8%	25%	---	6.3%	18.8%	---	---	---	25%
Fdn = 20	---	---	25%	---	25%	5%	5%	---	---	---	45%
Fcn = 14	---	---	21.4%	35.7%	7.1%	---	14.3%	---	7.1%	---	64.2%
Fbn = 26	4%	---	11.5%	7.7%	4%	---	4%	11.5%	11.5%	---	50%
Fan = 51	---	---	31.8%	9.8%	3.9%	3.9%	11.8%	9.8%	15.7%	---	29.4%
Dn = 40	---	---	12.5%	10%	15%	2.5%	15%	2.5%	22.5%	---	22.5%

*categories are not mutually exclusive and some artifacts are used on more than one material; therefore, rows may not total 100%.

### Plants

**Figure 3:**
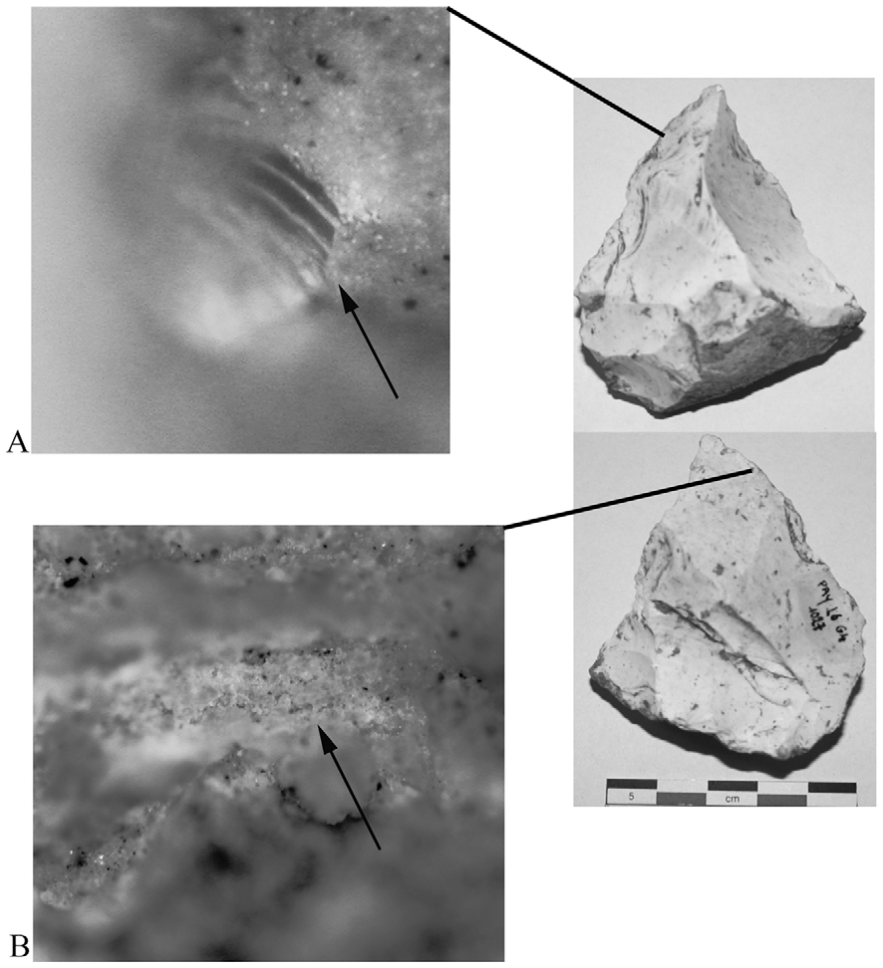
Wood processing tool. Payre L6 G4 1027, Layer Ga; A) scalariform perforation plate characteristic of birch (*Betula* sp.), original magnification 500x; B) wood tissue, original magnification 100x.

Woodworking activities (23/182 artifacts, 12.6%) were identified through diagnostic wood anatomy (tracheids, pitting, perforation plates, etc.) [30] and associated hard/high silica polish, striae and sometimes edge rounding. Woodworking is present in all levels except Ga; however, this level does have artifacts with undiagnostic plant tissue and hard/high silica polish. This evidence most likely represents woodworking. In two cases, diagnostic anatomy allowed more specific identification. Bordered pits on a flake from Level Fc indicates gymnosperm processing while a scalariform perforation plate on a sidescraper from Level Ga likely derives from birch (Fig. 3) [30]. An additional 23 artifacts showed plant processing but more specific identification of the type of plant was not possible.

**Figure 4:**
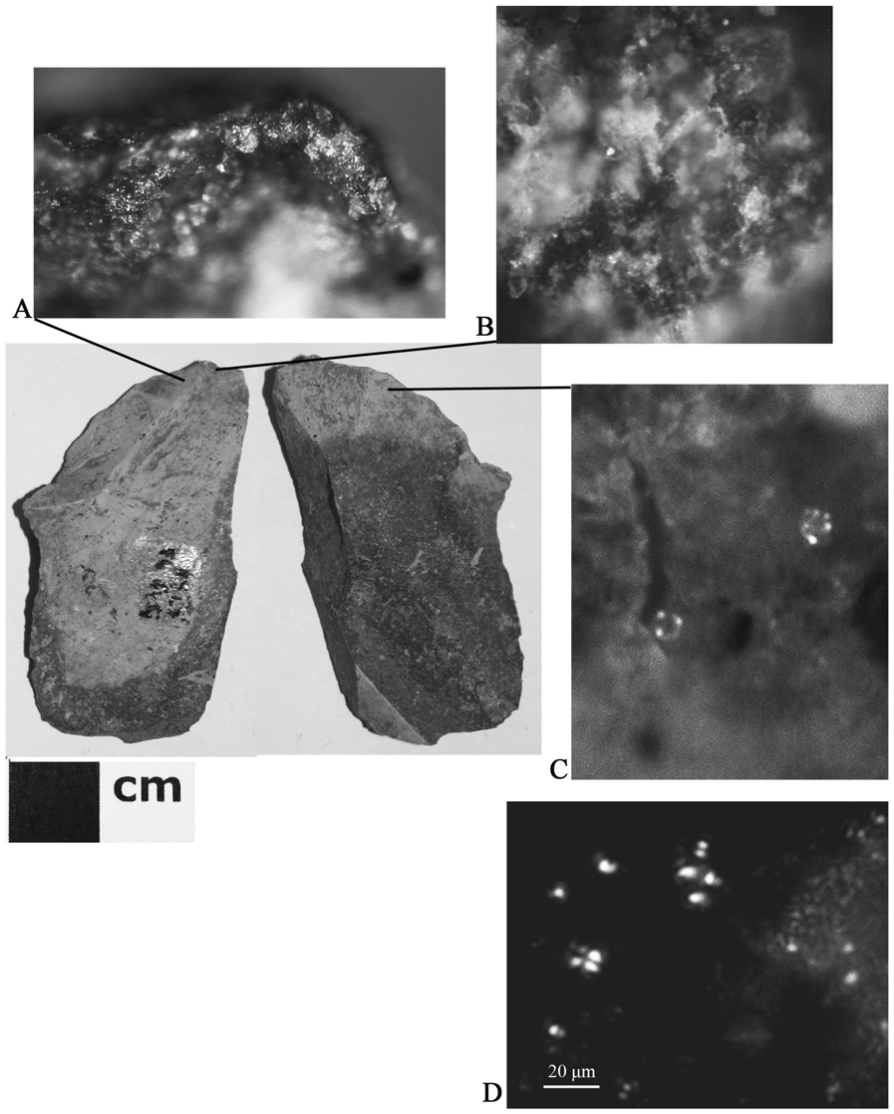
Starchy plant processing tool. Payre L7 F4 6451, Layer Fa; A) hard/high silica polish and edge rounding, original magnification 100x; B) macerated plant tissue, original magnification 100x; C) starch grains in situ, cross-polarized reflected light, original magnification 500x; D) starch grains extracted from tool, transmitted cross-polarized light, original magnification 500x.

Starchy plant processing (18/182 artifacts, 9.9%) was identified by the presence of starch grains exhibiting an extinction cross under cross-polarized light [45] and the co-occurrence of a suite other plant tissues and parts (Fig. 4) [44]. The starch grains observed ranged in size from 3–16 µm. Given their small size (many under 5 µm), putative starches were extracted from a sample of 10 artifacts for observation under transmitted light (500x) in order to confirm that they were not starch look-alikes such as conidia [44], [45]. Starch was confirmed on all ten artifacts. Many of the grains are 3–6 µm, spherical with a centric hilum, and lack lamellae. A smaller number are elliptical with an eccentric hilum and also lack lamellae. In addition, rectangular phytoliths were observed in extractions from two artifacts.

### Animals

As with plant processing, the identification of animal processing is most secure when supported by multiple lines of evidence [19], [46]. Evidence of mammal processing (31/182 artifacts, 17.6%) in the sample includes hair, skin, bone, muscle tissue and accompanying wear patterns (soft polish and striae). Skin and hair fragments on scrapers demonstrate hidescraping while hair, bone, and skin on unmodified flakes suggest animal butchery. Animal processing is present throughout the sequence at Payre indicating that this was a routine activity.

**Figure 5:**
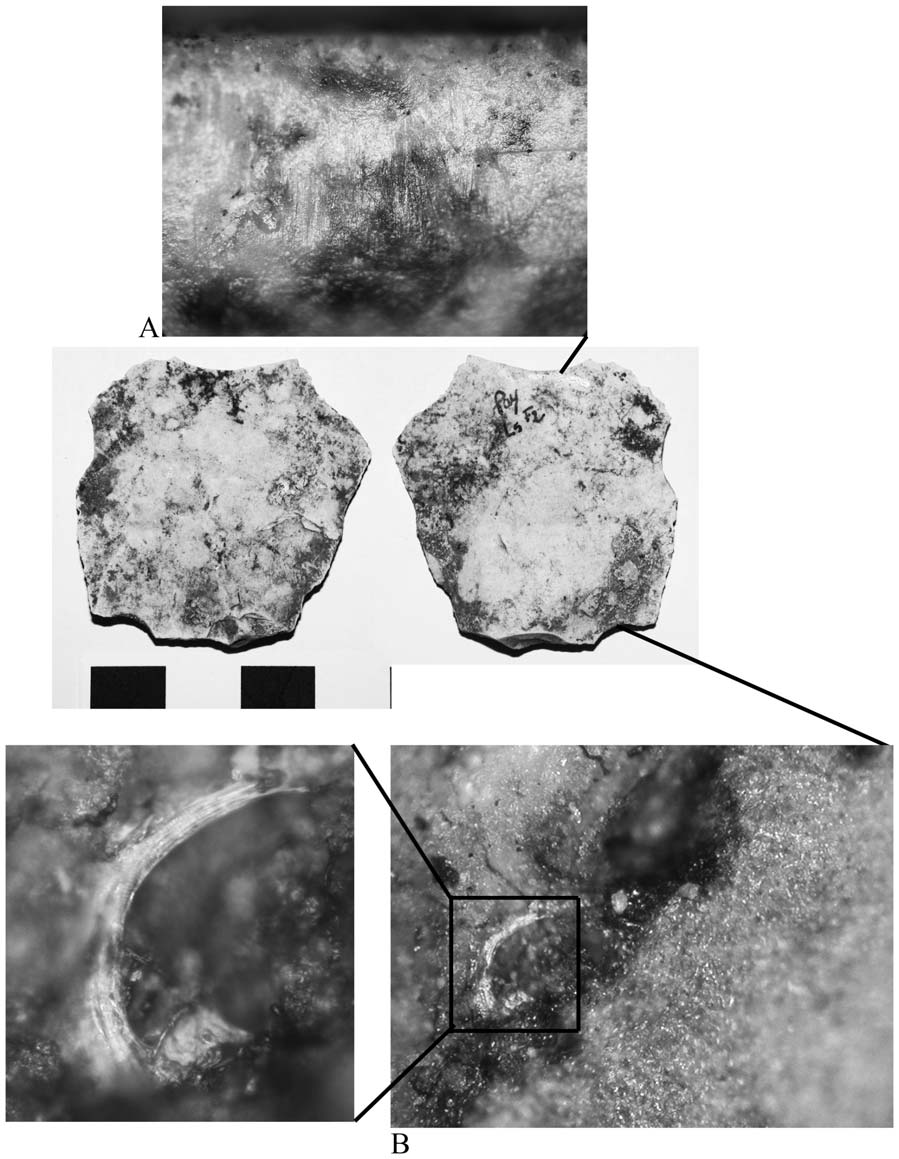
Fish processing tool. Payre L5 F2, Layer Fa; A) polishing with linear streaks characteristic of processing fish, original magnification 100x; B) fragment of a ctenoid fish scale, original magnification 100x.

Recent experiments involving scaling and butchering fish with stone tools [43] as well as clarification of use-wear patterns associated with fish processing [22] have provided new criteria for recognizing fish exploitation in the archaeological record. Use-wear patterns associated with fish include scalar edge scarring and randomly oriented streaks of dull, greasy polish. These traces, however, have often been viewed as ambiguous [23]. Högberg et al. [22] have recently used protein analysis to confirm that this use-wear pattern is indeed evidence of fish processing. At Payre, identification of fish processing was only made if characteristic wear patterns were accompanied by fish residues (Fig. 5). Fish residues identified included scale fragments, bone fragments, iridophores (pigment cells of the epidermis), and skeletal muscle. Fish processing first appears in Layer Fb (beginning of OIS 7) and continues through Fa and D (end of MIS 6/beginning of MIS 5). A total of 10 artifacts show evidence of fish processing. The lack of fish bones at Payre could be due to taphonomic bias or could suggest that fish processing took place off site while some fish processing tools were curated and returned to the site.

**Figure 6:**
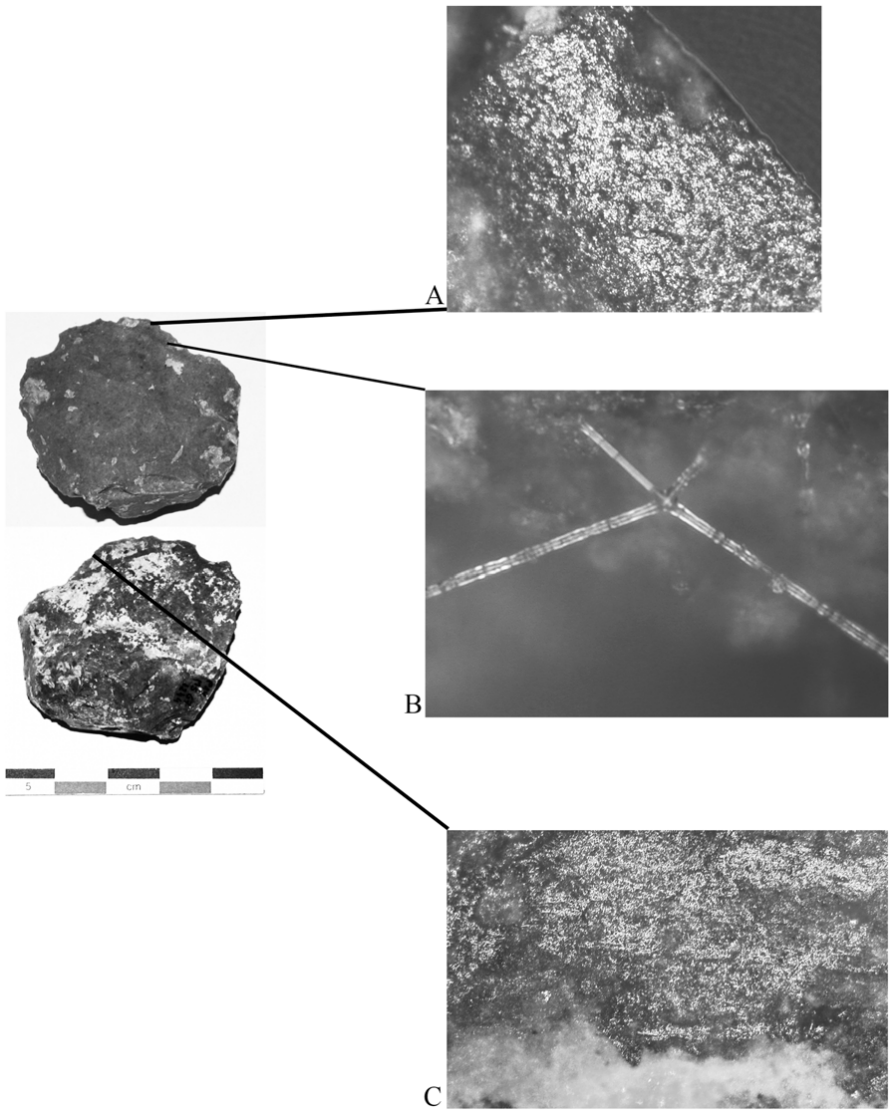
Bird processing tool. Payre M5 G7 1244, Layer Ga; A and C) soft polish, original magnification 100x; B) feather barbule fragment, original magnification 500x.

One artifact from layer Gb shows evidence of use of avian resources. This artifact has soft use-wear polish accompanied by feather barbules (Fig. 6). While this single example does not provide information about the frequency of use of avian resources, it does attest to their use [48].

## Discussion

### Patterns of Tool Use

The stone tools examined from Payre show a diversity of action and use-materials and suggest broad-based economic and subsistence activities. The discoid flaking method typical at Payre provides many useful cutting edges that appear to have been used whatever the size and shape of the artifacts. As has been observed elsewhere in the Middle Paleolithic (La Quina, Starosele, and Hohle Fels), there is no specific use associated with different tool types [19]–[21]. This corroborates earlier findings on convergent tools from Payre analyzed through macro-traces [49]. Furthermore, shape, presence of cortex, and size do not correlate with specific uses. At Payre, the artifacts are primarily made from local flint, but some is imported from as far as 60 km away, arriving as broken nodules or large flakes [15]. No specific technical behavior is observed on this rare or the local flint. The selection of stone tools for different activities thus appears to have been utilitarian and opportunistic. Tools are used for one part of their edges and not for their general form or location of retouch. Both retouched and unmodified edges are used frequently. The tools from Payre show a great variety of forms because retouch is not invasive and does not modify the general shape of tools. Resharpening is rare; therefore, individual sections of artifacts are functionally important, not the entire piece.

### Economic and subsistence activities

Woodworking is common in all levels at the site. Levels Gb/Ga (MIS 8/7) and D (MIS 6/5) formed in a temperate context when wood have been available in great quantities around the site [50]–[51]. Level F would have formed in a cooler context, but wood was still available near the site. It is impossible to predict precisely the types of wooden tools or objects that were being shaped through this activity, but the almost complete lack of evidence for hafting (with the exception of one scraper from Level Fa) suggests the manufacture of other types of wooden technology. Both microscopic and macroscopic analyses of convergent tools from Payre do not show any indication of hafted projectile points. Nevertheless, hunting is clearly attested by residue and use-wear evidence and faunal remains. One likely woodworking activity may therefore have been the construction of spears similar to those recovered from Schöningen [52].

The diet at Payre was quite diverse, including plants, large and small animals, fish, and possibly birds. Starchy plant processing first appears in level Ga and continues through the rest of the sequence. Given the large number of potentially edible species that would have been available to Neanderthals [5] and the lack of a sufficiently detailed comparative collection, we do not provide a more specific identification. The morphology of the stone tools used for starchy plant processing, unmodified flakes and scrapers, along with the use-wear patterns suggests a scraping or cutting motion. Since the two major categories of wild edible plant foods with significant quantities of starch are underground storage organs (USOs) and seeds, the most likely use was in the removal of the woody and unpalatable exterior of USOs. This evidence suggests that starchy USOs were a regular part of the Neanderthal diet at Payre from MIS 8/7 onwards. These results corroborate the recent evidence of starch grains in Neanderthal dental calculus at Spy and Shanidar [4] and demonstrate that Neanderthal consumption of plants was routine as early as MIS 8/7.

Animal processing at Payre includes both butchery and hideworking activities. Hair and skin fragments are found on a variety of retouched and unretouched tools. In addition to large animals attested to by skeletal remains (including *Cervus elaphus*, *Bos primigenius*, and *Equus ferus*, among others), fish and birds were also processed. Fishing and fowling are often used as markers of modern human behavior [53], despite their remains having been reported from numerous early hominin (as far back as 1.95 Ma) [54] and Neanderthal sites (see below). In fact, fishing is difficult to detect in the archaeological record for several reasons: 1) many coastal sites are lost due to rise in sea level; 2) fish bones are fragile and may be lost due to taphonomic processes; 3) many fish bones are small and may require specialized recovery techniques; and 4) the widespread assumption that fishing is a modern human behavior may lead investigators not to look for evidence in the first place [55]. The argument that Neanderthals did not fish has recently been bolstered by stable isotope research [56]–[57] that suggests that Neanderthal δC^13^ values do not match those of fish. This evidence must be treated with caution, however, as δC^13^ for fish can vary greatly, particularly from freshwater fish [58]–[59].

Sites with possible evidence of Neanderthal consumption of fish include Milan, Almada and Abreda Caves, Spain [60]–[61], Grotte XVI, France [62], Devil's Tower and Vanguard Cave, Gibraltar [63], Raj Cave, Poland [64], Grotta Maggiore, Italy [65], Ust-Kanskaya Cave, Siberia [66], and Figueira Brava Cave, Portugal [67]. Evidence at these sites includes the recovery of osteological remains, fish bones in association with hearths, and cut-marks on fish bones. At Payre, residues and use-wear indicative of fish are found in the absence of osteological remains. Fish may have been processed off-site (at local streams or rivers), and the tools returned to the site or fish may have been processed on site but the bones did not preserve. In Level Fa, all of the artifacts with fish residues are located in one square meter near the wall, a possible indicator of a specialized intrasite activity area. These results highlight the difficulty in recognizing fish consumption archaeologically and suggest that fish consumption by Neanderthals may be underrepresented. The growing list of sites with fish remains as well as the detection of fish processing in the absence of fish bones at a site further suggests that fish consumption should not be seen as exclusively in the domain of modern humans.

The evidence of bird exploitation at Payre is less clear, but still present. As with fish, the exploitation of birds is commonly portrayed as part of a larger broadening of the dietary niche associated with modern humans which gave them an advantage over Neanderthals [68]–[69]. Recent finds at Bolomor Cave, Spain, showing butchery and consumption of birds (*Aythya* sp., diving ducks) demonstrate that at least some Neanderthals hunted and ate birds [70]. At Bolomor, the evidence of consumption is straightforward and includes cut-marks, burning and human toothmarks. Bird remains are found at several other Neanderthal sites but their interpretation is seen as ambiguous. Depending on the size of the bird and the method of processing and cooking, evidence of human activity (cutmarks, human toothmarks) may be lacking. Furthermore, birds may represent more than just food. For example, at Fumane Cave in Italy, cutmarks and scraping on wing elements of birds of prey have been interpreted as evidence of the removal of feathers for ornamentation [71].

Osteological bird remains at Payre include *Tetrao tetrix* (black grouse), *Pyrrhocorax graculus* (alpine chough), *Corvus monedula* (jackdaw), and *Corvus corone* (carrion crow), but none of the bones display cutmarks. Previous research has suggested that the remains would have been brought into the cave by carnivores [13]. However, an endscraper from Level Ga exhibits soft polish and fragments of feather barbules indicating that it was used in processing bird tissue and feathers. While it is possible to potentially identify feather barbules to the Order level [34], isolated fragments may not preserve sufficient anatomical characteristics to do so [19] and therefore the taxon for the feather residues remains unknown. Two of the species of birds at Payre (black grouse and alpine chough) are also represented in the Fumane sample that may have been exploited for feathers. All four species at Payre are of low food value and, if they were introduced by humans, may have been of more interest for their feathers. At this point, however, this suggestion remains speculative.

The occupants of Payre were exploiting a wide range of materials. This fits with results of dental wear analysis of fauna that indicate a series of short-term occupation (level F) and longerterm occupations (level G and D) [12]. Faunal analysis on a regional scale (MIS 7 to MIS 3) indicates a far-sighted circulating model with occupations of various durations [72]. Payre would have been primarily served as a short-term seasonal occupation site over time.

The occupations at Payre all occur at the beginning of interglacial cycles. The climate was therefore temperate through the entire archaeological sequence. The location of the site on a promontory above the Rhone and Payre valleys would have provided easy access to a diverse range of resources. The topography of the area would have allowed access to multiple ecological zones, including plateau, slope terraces, valley floor, streams and rivers. While there is some variation in the range of resources exploited (starchy plants first appear in level Gb and fish in level Fb, bird confined to level Ga), all activities attested to by the functional analysis of stone tools at Payre appear in end of MIS 8/beginning of MIS 7. Occupations in both MIS 8/7 and MIS 6/5 show a similar pattern of use of a broad range of resources. However, it is unclear from the present data whether this pattern of resource exploitation was characteristic of all Neanderthal populations or whether it is unique to Payre. Some have suggested that a broader resource base could be tied to temperate conditions and that during colder conditions resources may have been more limited and subsistence more focused on large herbivores [6]. However, even during colder conditions, some plant foods remain available [5] and it is likely that Neanderthal diet varied according to what was available [73]. Nonetheless, the broad range of resources exploited at Payre certainly demonstrates that Neanderthals had the ability to access them.

### Conclusions

Neanderthals are often portrayed as specialized large game hunters who derived the vast majority of their diet from meat [74]. They are seen as having little interest in, or being incapable of acquiring, small game, fish, birds, or plants [75]. This view remains dominant in the field despite growing evidence to the contrary [4], [50], [60]–[61], [63], [70]. For example, it has been commonly accepted that Neanderthals could not hunt birds. New data from Bolomor Cave [70], Fumane [71] and now from Payre suggest that this view is not accurate. Furthermore, results from Payre now provide evidence that Neanderthals could acquire fish, an activity that is often seen as too advanced for Neanderthals [59]. Functional analysis of stone tools at Payre further bolster the case that Neanderthals had a broad-based diet that included starchy plants, large animals, fish, and possibly birds. The acquisition of fast-moving small prey items such as fish and birds are often seen as exclusively the domain of modern humans and their capture is often linked to a presumed cognitive superiority of modern humans [3]. The remains of birds and fish are fragile and often do not preserve as well as those of larger animals. This introduces a potential bias into the archaeological record in favor of large terrestrial game. Furthermore, as seen at Payre, the processing of these prey items may leave no archaeologically detectable trace on bone. As the results from Payre demonstrate, zooarchaeological analyses do not provide a perfect record of the activities at a site. In this case, the application of residue and use-wear analyses revealed activities that were otherwise not visible. The exploitation of plants, birds, and fish were all undetected by more traditional forms of analysis.

Neanderthals are often defined by their extinction. Because they went extinct, they must have been doing something wrong. However, as evidence continues to mount that shows that Neanderthals practiced what has been considered exclusively modern human behavior (plant consumption, fishing and fowling, ornamentation, etc.), it is important to remember that Neanderthals prospered for over 200,000 years. Our evidence suggests that they did this, in part, with a broad-based diet and economy that was already in place 125–250,000 years ago.
